# Probing intrinsic defects of aluminium-doped CuO thin films for solar cell applications†

**DOI:** 10.1039/d4ra06413e

**Published:** 2024-11-05

**Authors:** Adithya Prakash, Vikash Mishra, Mahesha M. G.

**Affiliations:** a Department of Physics, Manipal Institute of Technology, Manipal Academy of Higher Education 576104 India mahesha.mg@manipal.edu

## Abstract

Intrinsic defects in semiconductor thin films play a significant role in determining their optoelectronic properties. In this work, we investigated the impact of aluminium doping on the intrinsic defects and, thereby, the optoelectronic properties of CuO thin films deposited *via* spray pyrolysis. Doping considerably influenced the inherent defects of CuO thin films. Al^3+^ doping enhanced oxygen interstitial defects and suppressed oxygen vacancy defects. The presence of oxygen interstitials and an improvement in the crystallinity of the films resulted in favourable changes in the properties of the films. The observed modifications in the properties had a profound significance in improving the performance of CuO-based optoelectronic devices such as solar cells. Further, the ease of formation of oxygen interstitial defects compared to other possible defects and their favourable role in enhancing optoelectronic properties were confirmed through theoretical calculations. Thus, *via* comprehensive experimental and theoretical investigation, this study provides significant insights into the formation of defects and their influence on the properties of Al-doped CuO films.

## Abstract 2

Defect-engineered Al-doped CuO thin films deposited *via* chemical spray pyrolysis find application in solar cell absorbers.

## Graphical abstract

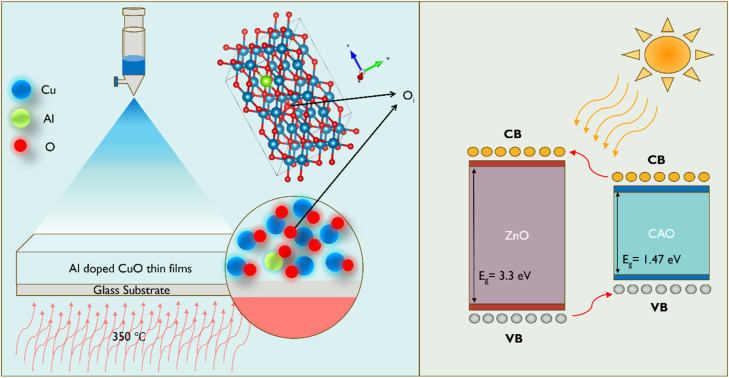

## 1. Introduction

Although many new materials, such as CIGS, perovskites and organic solar cells, are being studied for solar cell applications, scientific interest in CuO thin films persists. This retained interest is mainly because of their sustainability. The key issues with perovskite solar cells are their unsatisfactory stability^1^ and the toxicity of materials used for their production.^2^ Similarly, In and Ga elements in CIGS are costly and scarce and therefore unsuitable in the long run.^3^ Similar to the case of silicon, the natural abundance of copper and oxygen ensures their availability in the long run.^4^ This also makes them economically viable by reducing the cost of CuO thin-film solar cells compared to other materials available in the market. Additionally, both Cu and O are non-toxic, ensuring safety during synthesis. Moreover, as CuO is an oxide, numerous low-cost chemical deposition techniques are available for synthesizing CuO thin films. This further ensures the economic viability of CuO thin-film solar cells.^4^

While CuO thin-film solar cells have numerous advantages, it is important to understand their limitations. Despite their excellent optical properties, such as high absorbance and ideal bandgap tunability (1.4–2.1 eV), the efficiency of CuO thin-film solar cells remains low. High resistivity and poor mobility make charge separation difficult, reducing their efficiency. Thus, improving their electrical properties can offer a better performance of CuO solar cells. The intrinsic defects in CuO thin films have considerable influence on the properties of the films. While interstitial oxygen enhances p-type conductivity, oxygen vacancies enhance n-type conductivity in CuO thin films. Thus, the electrical properties of CuO thin films can be tuned by controlling these defects.

One of the most effective methods to influence the defects and properties of thin films is doping. In the case of aliovalent doping, charge compensation is achieved through the formation of defects, and the type of the defect favoured greatly depends on several factors, such as the ionic radius of dopant atoms and the charge of defects.^5^ In the case of donor defects, the additional charge of the donor atoms is compensated by the formation of metal cation vacancies or anion interstitials.^5^ Since copper vacancy and O_i_ enhance the conductivity of CuO thin films, choosing a donor dopant is wise. Hence, in this work, we have considered Al^3+^ as the dopant. In addition to this, the comparable ionic radii of Al^3+^ and Cu^2+^ make it suitable for doping in CuO with minimum lattice distortion.

Doping has been widely acknowledged for its significant impact on both the optical and electrical characteristics of CuO thin films. Despite numerous reports on the effects of various dopants on the properties of CuO thin films, a significant research gap on the influence of doping on inherent defects in CuO thin films and related properties persists. This study aims to address this gap by investigating interstitial oxygen defects, which are augmented by the introduction of the Al^3+^ dopant, in CuO thin films fabricated by spray pyrolysis.

## 2. Methodology

### 2.1 Experimental details

**Fig. 1:**
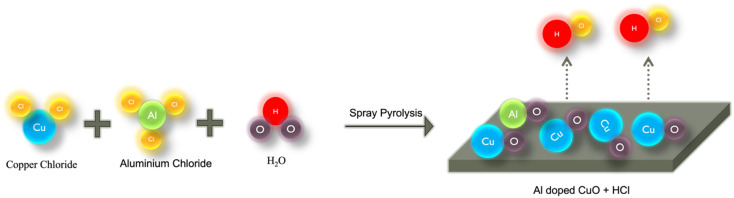
Schematic representation of CuO film formation *via* spray pyrolysis.

Thin films of Al-doped CuO were prepared by spray pyrolysis on a pre-cleaned soda-lime glass substrate. Analytical-grade CuCl_2_·2H_2_O and AlCl_3_ were used to prepare precursor solutions of desired doping concentrations. The glass substrates were cleaned using the standard cleaning procedure: ultrasonication in a soap solution followed by distilled water, acetone and IPA. The cleaned glass slides were then dried by purging N_2_ prior to CuO deposition. 0.15 M solutions of copper chloride and aluminium chloride were prepared using double-distilled water as the solvent in separate beakers. The stock solution was then prepared by mixing precisely measured volumes of both solutions depending on the required doping concentration. For each doped sample, 10 mL of the resultant stock solution was then loaded into a spray syringe. The Al doping concentrations employed were 0, 1, 2, 3, 4 and 5 at%, and the films were named CAO 0, CAO 1, CAO 2, CAO 3, CAO 4 and CAO 5, respectively. The thin films were then prepared by spraying this 10 mL stock solution onto the glass substrates maintained at 350 °C. The compressed air was used to create aerosols of the spray solutions while coming out of the nozzle. Upon contact with the substrate kept at elevated temperatures, these aerosols underwent a thermal reaction, resulting in the formation of films on the substrate. The volatile by-products were removed through an exhaust. The schematic representation of film formation on the substrate by pyrolysis is shown in Fig. 1. The spray rate, substrate temperature and precursor solution concentration were optimized and kept constant throughout the deposition process.

The properties of the prepared Al-doped CuO thin films were studied using different characterization techniques. The thicknesses of the samples, as measured using Dektak XT Profilometer, were in the range of 650–700 nm. The phase, crystallographic structure and preferred orientation of the films were identified using an X-ray diffractometer (Rigaku Miniflex-600 Diffractometer). The scans were performed in the range of 30° to 80° with a scan rate of 1° min^−1^ using a Cu-Kα X-ray source of wavelength 1.54 Å. Field Emission Scanning Electron Microscopy (FESEM) was employed to examine the surface morphology of the films using a Zeiss SEM EV01S. High-resolution micrographs of the film at different magnifications were obtained. The elemental composition and their distribution in the films were also determined using energy dispersive spectroscopy (EDS). The optical bandgap of the films was estimated using the absorbance spectra recorded on a SHIMADZU UV 1800 UV-vis Spectrophotometer. The structural and chemical properties of the films were investigated using Raman spectroscopy. Photoluminescence (PL) spectroscopy was adopted analyse the defects present in the samples. Both Raman and PL spectra were recorded using a Horiba LABRAM with an excitation source of 532 nm. X-ray Photoelectron Spectroscopy (XPS) was used to understand the surface chemistry of the films. The XPS spectra were obtained by irradiating the films with Al Kα radiation with a photon energy of 1486.6 eV using a PHI 5000 VersaProbe III. Lastly, the electrical properties of the films were evaluated based on Hall measurements carried out in the van der Pauw configuration.

### 2.2 Theoretical study

Quantum Espresso (QE) software was utilized for Density Functional Theory (DFT) computations, specifically by employing the plane-wave self-consistent field (PWscf) method.^6,7^ Heyd–Scuseria–Ernzerhof (HSE06), a popular and trustworthy hybrid functional^8–10^ was employed for all calculations, with the *α* (mixing parameter) value set at 25% and a screening parameter of 0.2 Å^−1^. The monoclinic space group *C*2/*c* was used for the calculations of pure and Al-doped CuO. We used pure and ∼1%, 3% and ∼5% Al-doped CuO crystal structures with a 3 × 3 × 3 supercell. A *k*-mesh of size 7 × 7 × 7 was employed within the first Brillouin zone.^11^ Self-consistent calculations were used with an energy convergence value of 4 × 10^−6^ eV, and forces per atom were reduced to 0.04 eV.^12^ SCAPS-1D software was used to simulate the *J*–*V* characteristics of the device with CuO as the p-type layer.^13,14^

## 3. Results and discussions

### 3.1 Structural characterization

**Fig. 2:**
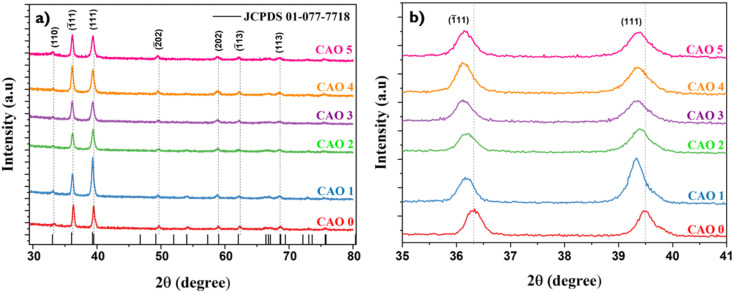
(a) X-ray diffractograms of CAO thin films; (b) shift in the peak positions of their (111) and (1̄11) reflection planes.

The impact of Al doping on the crystalline structure of CuO thin films was assessed *via* XRD. Fig. 2a illustrates the diffractograms of the films. All films exhibited consistent diffraction patterns characteristic of the monoclinic phase of CuO, with all observed peaks matching the standard JCPDS card (01-077-7718). No additional peaks corresponding to secondary phases of copper oxide or aluminium oxide were detected up to 5 at% doping, confirming the phase purity of the samples. This suggests that Al could successfully substitute Cu in the CuO lattice without causing major structural distortions or forming additional phases. Fig. 2b shows the change in position of two major peaks in the different samples. The incorporation of Al^3+^, which is a smaller ion (ionic radius: 0.53 Å) than Cu^2+^ (ionic radius: 0.73 Å), should ideally result in lattice contraction, leading to a shift in the XRD peaks to higher diffraction angles. Interestingly, the positions of the diffraction peaks shifted towards lower angles after Al doping. This anomaly in the peak shift can be due to the formation of O_i_ defects or strain induced in the CuO lattice after doping. A similar shift in the XRD peaks toward lower angles after Al doping has also been reported by M Arfan *et al.*^15^ The incorporation of Al^3+^ disrupts the charge neutrality of the system, and as a result, defects like O_i_ are created. Moreover, the strain introduced by doping can promote the formation of oxygen interstitial defects. When oxygen atoms occupy the interstitial sites, they introduce additional repulsive interactions with neighbouring oxygen atoms, thereby leading to lattice expansion. The peak shift observed in the diffraction pattern might be due to this lattice expansion.

The lattice expansion was further evident from the increase in lattice parameters. The relationship between the lattice parameters (*a*, *b*, *c*), Miller indices (*h*, *k*, *l*) and interfacial angle *β* of monoclinic CuO with interplanar distance *d*_*hkl*_ is given below.1
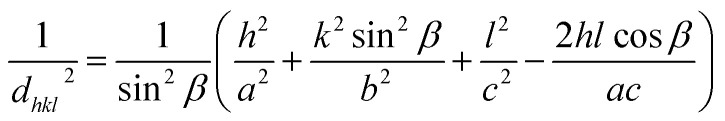


The lattice parameters of the prepared films were calculated by unit cell software, and the obtained values are given in Table 1. Doping led to an increase in lattice parameters. However, the values did not increase consistently. This is because of the interplay between lattice compression due to the smaller ionic radius of Al^3+^ and O–O bond stretching caused by O_i_ formation. A deviation in trend was observed for the 1 at% Al-doped sample. The CAO 1 sample showed also a decrease in cell volume and the *c* parameter. This may be due to the relatively lower concentration of Al doping in the sample. As the doping concentration is the lowest in this sample, the strain developed would be less than in other samples, which might be the reason for the observed deviation.

**Table 1:** Structural and optical properties of CAO films

Al dopant concentration (at%)	0	1	2	3	4	5
*a* (Å)	4.574	4.672	4.656	4.602	4.658	4.666
*b* (Å)	3.370	3.374	3.383	3.361	3.360	3.362
*c* (Å)	5.070	4.897	5.068	5.101	5.080	5.079
*V*_cell_ (Å^3^)	77.45	76.57	78.83	77.91	78.48	78.65
*β* (degrees)	97.78	97.39	99.15	99.11	99.21	99.25
Crystallite size (nm)	16 ± 1	28 ± 3	24 ± 4	8 ± 2	20 ± 4	20 ± 2
Dislocation density (×10^16^ m^−2^)	0.40	0.12	0.17	1.54	0.26	0.22
No. of crystallites (×10^18^ m^−2^)	0.12	0.02	0.03	1.24	0.11	0.12
TC_111_	0.85	1.17	1.04	0.97	0.92	0.97
TC_1̄11_	1.14	0.83	0.95	1.03	1.07	1.03
Band gap (eV)	1.50	1.45	1.48	1.54	1.46	1.47

A shift in the preferred orientation and variations in the crystallinity of the samples were observed after doping. The diffractogram of the films showed two sharp and intense peaks for all samples at approximately 36° and 39°, indicating that the preferred direction of grain growth was along the (1̄11) and (111) reflection planes. If *I*_*hkl*_ and *I*_0*hkl*_ represent the observed and standard intensity (from JCPDS) of *hkl* plane,2
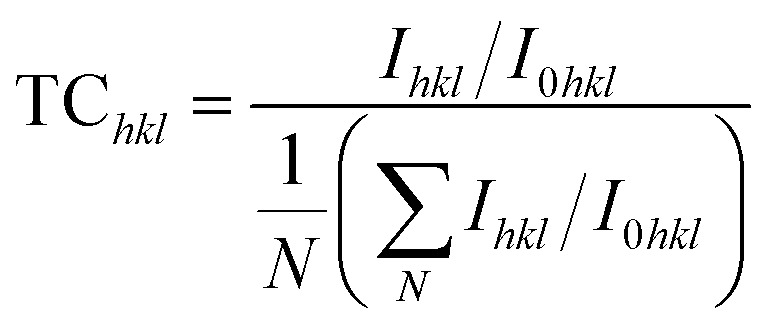


A value of TC > 1 indicates abundant crystallite growth in that particular direction, whereas a value of TC < 1 indicates limited growth in the given direction. The calculated TC_*hkl*_ values of (1̄11) and (111) given in Table 1 indicate a shift in growth orientation from (1̄11) to (111) at lower doping concentrations of 1 and 2 at%. This shift in the preferred orientation of crystal growth suggests that Al doping influences the growth dynamics of CuO thin films. At lower levels of doping, as the lattice strain developed might be less, the dopant showed a significant influence on the growth orientation. On the other hand, at higher doping concentrations, the balance between dopant incorporation and lattice strain is disrupted, resulting in a change in crystal growth orientation.

The size-strain plot was employed to calculate the crystallite size (*D*) of the samples, as given below.3
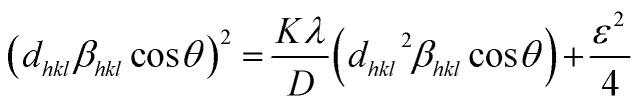
where *θ* is the diffraction angle, *λ* is the wavelength of the X-ray, *β*_*hkl*_ is the Full Width at Half-Maximum (FWHM) and *K* is the shape factor. The obtained *D* values of the films are presented in Table 1. The crystallite size of the films increased with Al doping; the 1 at% Al-doped sample showed the largest crystallite size of 28 nm. With a further increase in doping, the crystallite size decreased. An abrupt reduction in crystallite size was observed in the 3 at% Al-doped samples, with a drop in crystallite size to 8 nm. This might be due to restricted grain growth because of the strain developed during the growth process.

During the growth of films, disruptions in the regular arrangement of atoms lead to dislocations, and the number of such dislocations per unit volume is known as dislocation density (*δ*), which can be calculated as4
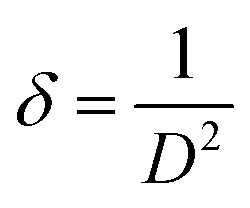


The calculated value of dislocation density was the highest for the 3 at% doped sample, indicating the highest concentration of dislocations produced in this film after doping. The density of crystallites is given by5
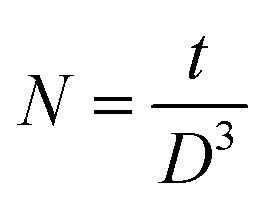
where *t* is the thickness of the films. The calculation revealed the lowest number of crystallites for the CAO 1 sample, suggesting that Al doping promotes crystallite growth, leading to larger crystallite sizes.

To understand the stability of the prepared films, the formation energy (*E*_f_) of the pristine and doped films with and without intrinsic defects was obtained through DFT calculations, and the values are given in Table 2. The defect-free undoped film had an *E*_f_ of −1.24 which rose to −0.08 after doping 1 at% Al. Higher doping concentrations subsequently led to positive formation energies. This suggests that a higher level of Al doping in CuO is not thermodynamically favourable and may result in unstable films. Experimental results also showed concurrent results; films with dopant levels above 5 at% were highly unstable and therefore, have not been included in this study. However, unlike the theoretical results, the films remained stable up to 5 at% Al doping. This might be because of the use of high temperatures during the deposition process, which provides enough energy to overcome the energy barrier associated with compound formation. Beyond 5 at% doping, this energy obtained from the heated substrate might not be sufficient to overcome the energy barrier, thereby resulting in highly unstable films.

**Table 2:** Calculated formation energy (*E*_f_) of pure and doped CuO with and without intrinsic defects

Al doping concentration (at%)	Formation energy (eV)				
	Without defects	With V_O_ defects	With O_i_ defects	With V_Cu_ defects	With Cu_i_ defects
0	−1.24	+1.20	+0.92	+2.10	+2.15
1	−0.08	+1.12	+1.02	+1.27	+1.32
3	+1.26	+1.56	+1.32	+1.40	+1.43
5	+1.37	+1.65	+1.68	+1.23	+1.25

The energy barrier that must be overcome for a defect to form, migrate, or interact within a material is called activation energy (*E*_a_). The least *E*_a_ of 0.06 was observed for CAO 3. This indicates that O_i_ defects were readily formed in CAO 3 compared to other films. However, such a high concentration of O_i_ defects can seriously impact the crystallite size of the film. In high amounts, these defects act as pinning sites and restrict the growth of crystallites, leading to finer grains. This explains the abrupt reduction in the crystallite size of the CAO 3 sample.

### 3.2 Morphological characterization and elemental analysis

**Fig. 3:**
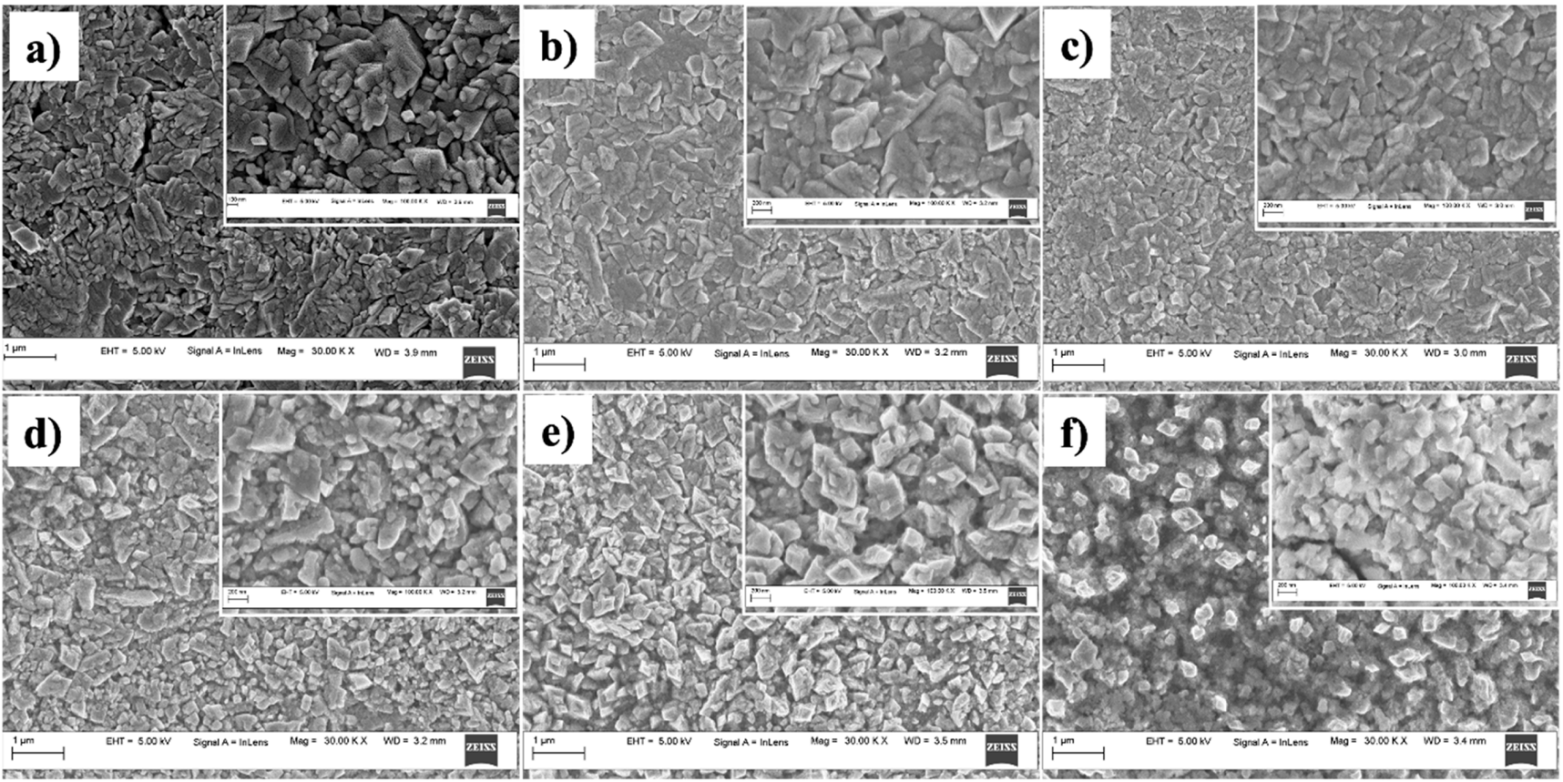
FESEM micrographs of (a) CAO 0, (b) CAO 1, (c) CAO 2, (d) CAO 3, (e) CAO 4 and (f) CAO 5 thin films.

The surface morphological features of the prepared films were analysed using the FESEM images presented in Fig. 3. All the films displayed high uniformity across the sample surface, with no visible variations or non-uniform growth. This indicates that Al doping did not induce any irregularities in film growth that led to clustering or non-uniformity of the films. In addition to this, the films showed no signs of cracks or discontinuities, which confirms the good quality of the films. The surface morphology of the Al-doped films closely resembled that of the undoped CuO films. All the films had similar flake-like morphology with more or less cube-shaped grains. The SEM images showed that the films contained grains of different sizes. This may be due to the difference in growth kinetics at different regions of the films depending on the number of dopant atoms. From the high-resolution images, it is difficult to assign perfect grain boundaries in these films. This may be due to the coalescence of smaller grains during grain growth, leading to larger structures. This suggests that Al doping promotes the growth of larger grains. The micrograph of CAO 3 shows a higher concentration of smaller grains, which is also observed in the high-resolution image.

The elemental composition of the films was determined by EDS, and the quantified amounts of each element are detailed in Table 3. The EDAX spectra revealed peaks indicative of Al, O, and Cu atoms, confirming their presence in the films. The elemental analysis suggests an increase in oxygen content in the films after doping. Moreover, the consistent decrease in Cu content and concurrent increase in Al content imply the substitution of Cu atoms by Al atoms within the films. The percentage difference between the ionic radii of the host and dopant atoms was calculated to find the substitution probability of Cu^2+^ by Al^3+^. The percentage difference (*D*_r_) between the radius of the dopant atom (*R*_d_) (Al^3+^) and the potential host cation (*R*_h_) (Cu^2+^) was calculated as^16^6
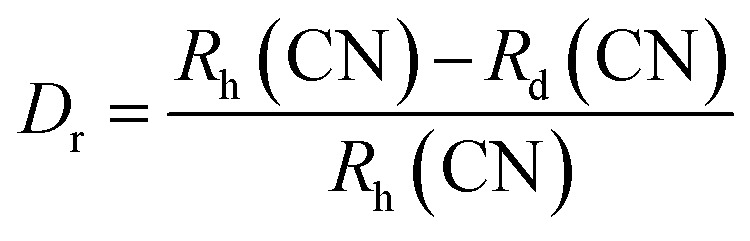
where CN is the coordination number. The CN of Cu^2+^ in CuO was 4, while that of Al^3+^ in Al_2_O_3_ was 6 (the secondary phase observed in XRD at higher doping concentrations was that of Al_2_O_3_). The ionic radius of 4-coordinated Cu^2+^ was 0.71 Å, while that of 6-coordinated Al^3+^ was 0.68 Å. The percentage radius difference calculated was found to be 4.9%, which is much lower than the acceptable 30%, further confirming the substitution of Cu^2+^ by Al^3+^.

**Table 3:** Elemental composition of different films (in the last column, the values in the bracket indicate the nominal ratio)

Sample	Cu (at%)	O (at%)	Al (at%)	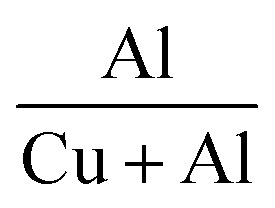
CAO 0	46.21	53.79	0	0
CAO 1	41.37	58.07	0.6	0.01 (0.02)
CAO 2	40.68	58.43	0.8	0.02 (0.04)
CAO 3	39.81	58.61	1.58	0.04 (0.06)
CAO 4	38.63	59.43	1.93	0.05 (0.08)
CAO 5	35.54	61.68	2.79	0.07 (0.10)

**Fig. 4:**
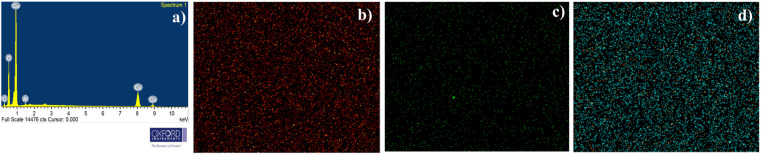
Representative (a) EDAX spectrum and elemental mapping of (b) O, (c) Al, and (d) Cu of the CAO 1 sample.

The quantity of Cu atoms substituted by Al atoms was calculated as the ratio of the obtained value for Al to the total content of Al + Cu. The ratio of Al/(Al + Cu) indicated that the fraction of Cu atoms substituted by Al atoms was lower than the expected nominal value, possibly due to the difference in reaction kinetics between the Al and Cu salts. Fig. 4 displays the elemental mapping of the CAO 1 film, illustrating the uniform distribution of all elements throughout the film. This further rule out the formation of elemental clusters that can affect film quality.

### 3.3 UV-vis spectroscopic analysis

**Fig. 5:**
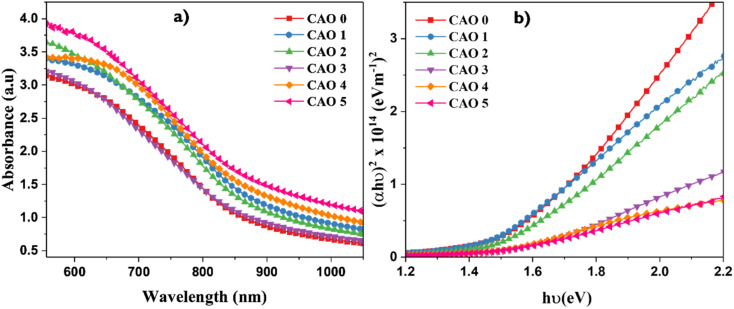
(a) Absorbance spectra and (b) Tauc plots of CAO films.

The optical properties of the CAO thin films were investigated by UV-vis spectroscopic analysis. The characteristic absorption spectrum is given in Fig. 5a. All the films showed high absorbance in the visible region, with a gradual decrease at higher wavelengths. The absorbance edges of the films were observed in the IR region and showed a red shift as the CuO films were doped with Al. This can be attributed to the enhanced dimensions *i.e.* larger crystallites of the CuO nanoparticles with the introduction of Al dopants. Additionally, the absorbance of the films increased with Al doping. This indicates that the light absorption capacity of the CuO thin films was improved by Al doping. All the films exhibited high absorption coefficient values in the range of 10^6^ m^−1^, probably due to the high density of crystallites in these films, as observed in the FESEM images. The absorption of the films also depends on the morphology of the films. The flake-like morphology of the films improves the scattering of light and thereby increases light absorption.^4^

The direct band gap (*E*_g_) of CAO films was estimated using the Tauc relationship^17^ given below.

7 *αhν* = *B*(*hν* − *E*_g_)^1/2^

where *hν* is the incident photon energy, *α* is the absorption coefficient and *B* is the energy-dependent constant. Fig. 5b shows the Tauc plots of the films, and Table 1 lists the calculated direct bandgaps of both pure and doped films. The bandgap of the films decreased marginally with doping. Initially, the pristine film had a bandgap of 1.50 eV, which decreased to 1.45 eV after doping with 1 at% Al. The bandgap variation shows a direct relationship with the size of crystallites in the respective films. As the crystallite size increased with doping, the bandgap decreased. The size effect of the bandgap was further verified by the increased bandgap of the CAO 3 film corresponding to its smallest crystallite size.

The simulated optical absorption spectra showed variations in the optical bandgaps of Al-doped CuO (see Fig. S1†). To investigate the impact of Al doping on the electronic properties of CuO, we computed the total density of states in CAO 0, CAO 1, CAO 3, and CAO 5 (a) without defects and (b) with O_i_ defects (see Fig. S2†). In the case of defect-free structures, the electronic bandgap decreased with an increase in Al doping in CuO. In the presence of O_i_ defects, the electronic bandgap also decreased with Al doping below 3% and matched well with the experimental values.

### 3.4 Raman analysis

Raman spectroscopy is a powerful technique to understand the structural defects and microstructural properties of materials at the nanoscale. It is sensitive to the local arrangement of atoms and the vibrations of the molecule. Hence, it can detect even minor phases present in films that are not detectable by XRD. It also provides information about the structure and bonds in materials. CuO is a p-type semiconductor that crystallizes in the monoclinic system. They belong to the space group of C^6^_2h_ with two molecules in the primitive unit cell. Each Cu ion is located in a plane formed by two equidistant oxygen atoms. The vibrational modes of CuO are given by,^18^

8 *Γ* = 4A_u_ + 5B_u_ + A_g_ + 2B_g_

**Fig. 6:**
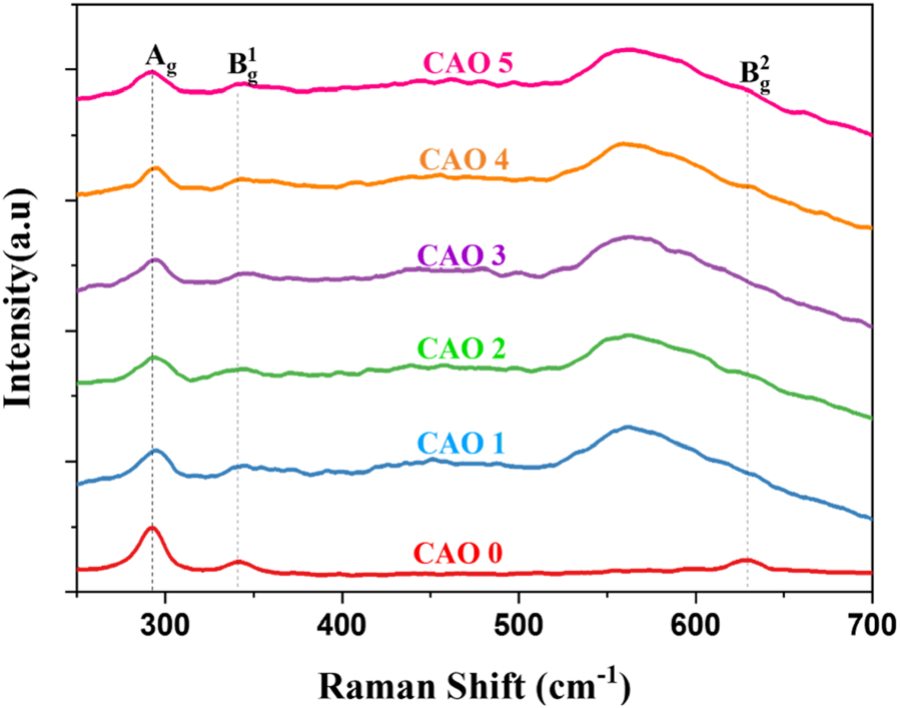
Raman spectra of spray-deposited CAO films.

Out of these different modes, only three are Raman-active (A_g_ + 2B_g_) and six are IR-active (4A_u_ + 5B_u_). In all the Raman modes, only oxygen atoms vibrate, whereas the Cu atoms are stationary. The vibrations of O atoms along the crystallographic *b*-axis contribute to the A_g_ mode, whereas the vibrations of O atoms along the *a*-axis result in the B_1g_ mode. The B_2g_ mode arises from the vibrations of O atoms perpendicular to both *a* and *b* axes.^19^Fig. 6 shows the Raman spectra of the prepared CAO thin films. All the samples showed the presence of all three Raman active modes. In the pristine samples, the different modes were observed at 293 (A_g_), 342 (B_1g_) and 628 cm^−1^ (B_2g_). These values are in good agreement with the previously reported values.^20^

After doping Al atoms into the CuO thin films, noticeable shifts in peak positions and changes in peak intensities were observed. Specifically, the peaks exhibited broadening and decreased intensity following doping, which are usually caused by crystal defects.^18^ The peak corresponding to the B_1g_ mode became broader and nearly diminished in the doped samples. On the other hand, the B_2g_ mode peak became broader and much more pronounced. The variation in intensity and broadening of the B_2g_ modes are due to the lattice changes introduced by the interstitial oxygen atoms. These interstitial atoms distort the structure near the defects and create a weak bond with the neighbouring lattice oxygen.^21^ This extra bond causes lattice expansion, which causes these changes in the B_2g_ mode peak as this mode corresponds to the symmetric stretching mode of O atoms.^22^

### 3.5 Photoluminescence study

**Fig. 7:**
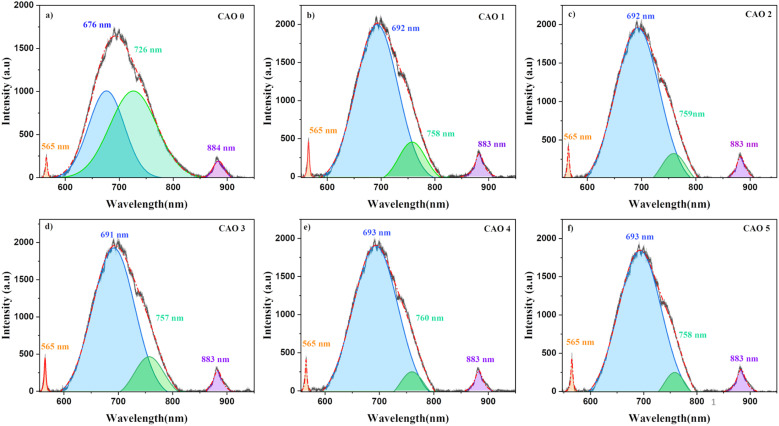
Photoluminescence spectra of (a) CAO 0, (b) CAO 1, (c) CAO 2, (d) CAO 3, (e) CAO 4 and (f) CAO 5 films.

PL spectroscopy is an important technique for investigating various electronic transitions and their influence on the optoelectronic properties of semiconductors. Thin films fabricated by deposition methods are often associated with different types of inherent defects, which can introduce defect levels within the bandgap. The electronic transitions between these levels produce emissions that are specific to the system. The defects in the prepared CuO and Al-doped CuO films were analysed using PL spectroscopy. The obtained PL spectra of all the films using an excitation wavelength of 532 nm are depicted in Fig. 7. The films showed the presence of three peaks, including two minor peaks at 565 nm (2.2 eV) and 883 nm (1.4 eV), and a prominent broad peak at around 600–800 nm. The deconvolution of this broad peak revealed the presence of two strong emissions at 676 and 726 nm, which red-shifted to 692 and 758 nm, respectively, in the doped samples.

The peaks in PL spectra can arise from different defect-related emissions. The most common point defects in CuO are vacancies, antisites and interstitial defects *i.e.*, oxygen vacancies (V_O_) or copper vacancies (V_Cu_) and oxygen interstitials (O_i_) or copper interstitials (Cu_i_). The presence of oxygen at Cu sites results in Cu antisite defects (O_Cu_), whereas Cu in oxygen lattice sites forms oxygen antisite (Cu_O_) defects. However, several first-principles calculations based on the density functional theory (DFT) have shown that the formation of these defects largely depends on the growth environment of the films.^21^ V_Cu_ is the most energetically favoured defect in CuO under O-rich conditions. This creates shallow acceptor levels above the valence band (VB). Moreover, the presence of V_Cu_ leads to an enrichment in oxygen content, creating O_i_ in the films. The formation energy of V_O_ is much higher than the formation energies of both V_Cu_ and O_i_. Hence, the formation of V_O_ is very limited.^23^ On the other hand, under Cu-rich conditions, the formation energy of V_O_ is significantly reduced and that of V_Cu_ is much higher, causing significantly higher concentrations of V_O_ defects.^24^ This difference in defect formation can also affect the electrical properties of CuO films. A higher concentration of V_Cu_ leads to a greater number of oxygen ions that accept electrons, leading to p-type conductivity. However, when V_O_ dominates, Cu_i_ donates electrons, resulting in n-type conductivity.^21^

**Fig. 8:**
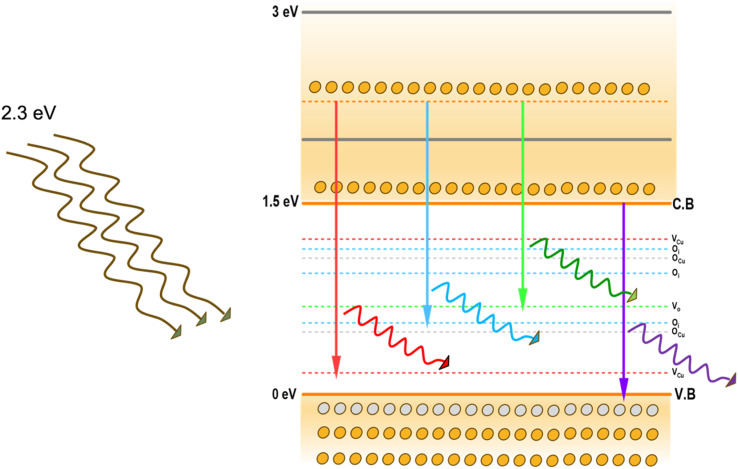
Schematic of the possible emission processes in CuO.

In spray deposition, since the hot substrates are exposed to the atmospheric air during and after deposition, the CuO films are usually O-rich in nature. The higher percentage of oxygen observed in the EDS analysis strongly supports this hypothesis. Therefore, hereafter, our discussion focuses on emissions in O-rich conditions. Aleksander Zivkovic *et al.*^21^ studied the positions of the energy levels of these defects in CuO using DFT calculations. V_Cu_ introduces two transition levels, including a shallow level at 0.17 eV above the VB and another one at 0.28 eV below the conduction band (CB). A deep transition level is created by O_i_ at 1.17 eV above the VB. The acceptor levels of O_Cu_ are located at 0.49 eV and 1.07 eV above the VB. The position of the V_O_ transition level in O-poor conditions is at 0.69 eV above the VB. In addition to these isolated defects, defects can also be formed in pairs with significantly reduced formation energy. The formation of O_i_ defects with already existing V_Cu_ defects produces O_i_ transition levels at 0.56 and 0.95 eV above the VB. The representative energy level diagrams of CuO thin films based on these values are given in Fig. 8.

**Fig. 9:**
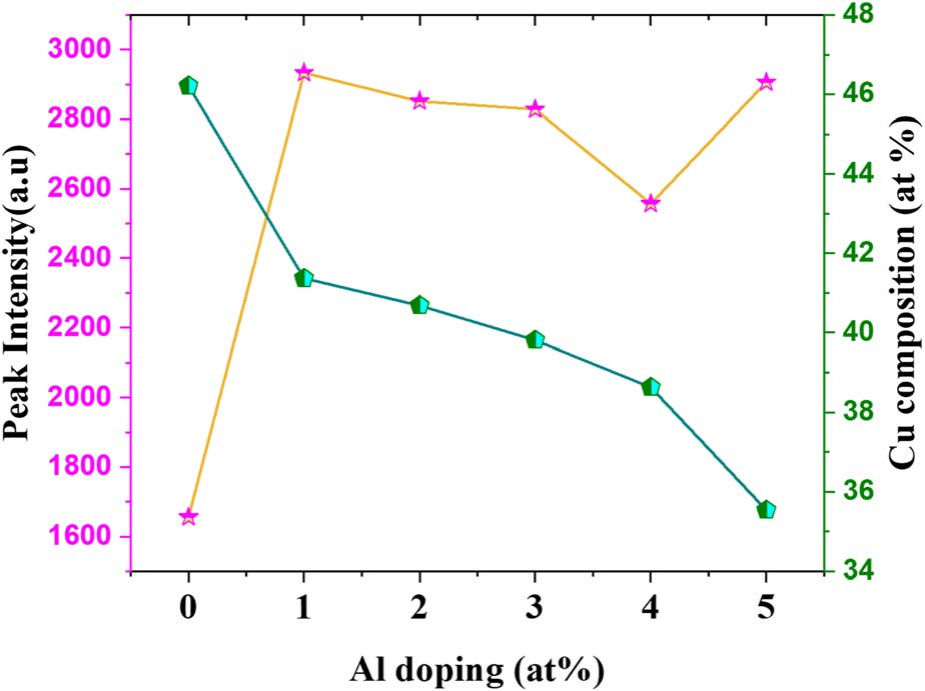
Variation in V_Cu_ peak intensity with Al doping concentration.

Based on this energy diagram, the transitions corresponding to various emission peaks observed in the PL spectra of the as-prepared samples are explained. The peak at 883 nm indicated the transition from the bottom of CB to the top of VB, which is the band-edge emission. All the other three transitions observed in the spectra were defect-related emissions. Usually, defect-related emissions have energy less than the bandgap. However, in this case, we observed defect emissions with energies greater than the bandgap. This can be explained by the excitation energy of the source. The PL spectra of all CAO samples were obtained using an excitation source of 532 nm (2.3 eV), which is much higher than the bandgaps of the samples. Thus, when the films were illuminated using a higher energy source, the electrons from the VB were excited to energy levels above the valence band. The transition from this excited energy level to the defect level resulted in the defect emissions; therefore, the energy corresponding to these defect emissions is greater than that of the bandgap. The emission at 726 nm arises from the transition from the excited energy level to the V_O_ level, while the emission at 676 nm is attributed to the transition from the excited state to O_i_ levels.^18,25^ The peak observed at 565 nm corresponds to the transition between the excited energy level and the V_Cu_ levels.^21^ Though some reports mention that V_Cu_ does not produce any PL emissions as it is the most stable defect,^26^ the probability of V_Cu_ emissions in this case cannot be eliminated. Fig. 9 illustrates the correlation between the copper content measured by EDS and the area of the 565 nm photoluminescence peak. An inverse relationship between Cu content and the intensity of the V_Cu_ emission was observed. Moreover, the reduction in Cu content was more than the content of Al added. This further supports the possibility of the formation of V_Cu_ defects. However, the much smaller peak area of this peak than other emission peaks in the PL spectra indicates that the probability of V_Cu_-related emissions is less compared to other defects.

The peak area and position of the two major defect peaks related to V_O_ and O_i_ showed considerable variations after doping. The PL spectra illustrated red-shifted peaks because of the impact of Al doping on defect formation. The areas of the peaks corresponding to emissions related to both V_O_ and O_i_ defects were observed to be nearly equal in the pristine sample, suggesting that both V_O_ and O_i_ co-existed in these CuO thin films. However, after doping, the area of the peak related to V_O_ decreased considerably and the area of the peak related to O_i_ increased. This indicates that Al doping suppresses the formation of V_O_ and improves the number of O_i_ in the doped films. The enhancement of O_i_ can further influence the electrical properties of the films, which is discussed later.

The formation energies tabulated in Table 2 exemplify that among the different intrinsic defects, O_i_ defects have the least positive values up to 3 at% doping. This indicates that among the different defects, O_i_ defects are easy to form. The other possible defects are V_O_ followed by V_Cu_. Based on calculations, the least possibility of formation was for Cu_i_, as evident from its highest formation energy compared with other defects. This explains the defect-related peaks and their intensity in the PL spectra. The maximum intensity was shown by the O_i_ peaks followed by V_O_ and V_Cu_, in accordance with the theoretical calculations. Further, the absence of Cu_i_-related peaks in the PL spectra substantiates the theoretical calculations.

### 3.6 X-ray photoelectron spectroscopy

**Fig. 10:**
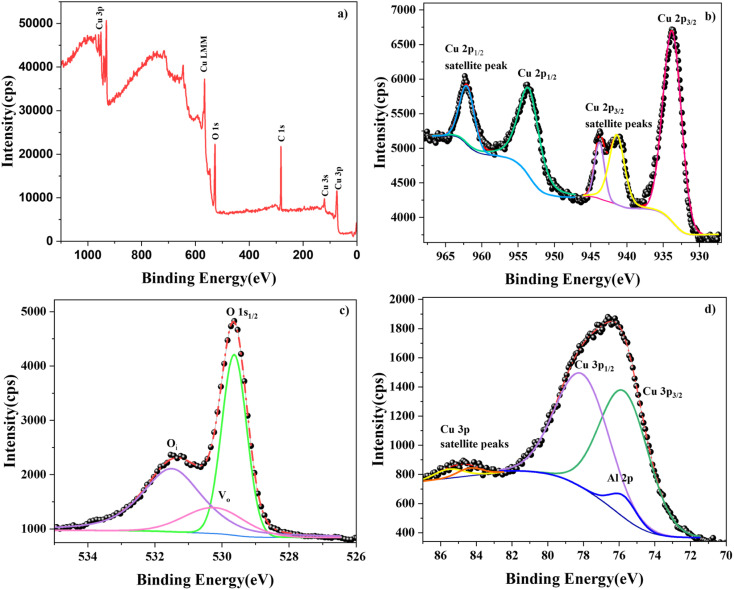
(a) XPS survey and core spectra of (b) Cu 2p, (c) O 1s, and (d) Cu 3p–Al 2p subshells.

XPS was used to identify the chemical state of the elements in the prepared films. Fig. 10 displays the survey and core spectra of the 1 at% Al-doped sample. The different peaks in the survey spectrum (Fig. 10a) were identified, and the presence of elements Cu, O, and C was confirmed. The peak corresponding to dopant Al could not be distinguished in the survey spectrum. This is because the positions of the Cu 3p and Al 2p peaks fall in the same binding energy region as Al, making it difficult to resolve the peaks.^27^ All the core spectra were carbon-corrected (284.8 eV) before fitting to avoid peak shift due to charging effects. The peak parameters of the peaks in the core spectra were identified after deconvolution using Shirley background.

In Fig. 10b, the deconvoluted Cu 2p core spectrum reveals five peaks including both satellite and main peaks. The major peaks observed at 933.77 eV and 953.50 eV correspond to Cu 2p3/2 and Cu 2p1/2, respectively, consistent with values reported in the literature.^28^ However, a nominal shift towards the higher-binding-energy region was observed compared with those of the pristine sample.^29^ This blue shift of the binding energy can be due to the influence of the Al dopant atoms. As the chemical environment around the Cu atom changed due to the substitutional effect of Al atoms, including lattice distortion and defects, the binding energy of the Cu 2p peaks showed a slight shift.^30^ The spin energy separation of the Cu 2p doublet was found to be 19.73 eV with a peak area ratio of 1.6, confirming the +2 oxidation state of Cu.^31^ The divalent oxidation state of Cu ions was further verified by the presence of satellite peaks near the higher binding energies of the Cu 2p doublet peaks.^32^

Further, the influence of Al integration in the CuO lattice was analysed using the O 1s core spectrum. The deconvoluted O 1s spectrum of CAO 1 is depicted in Fig. 10c. The prominent high-intensity peak at 529.64 eV is ascribed to the O^2−^ ions of the metal–oxygen bond, where the oxygen atoms are bonded to Cu^2+^ ions in the lattice.^33^ A shift in binding energy to higher values was observed in this case as well, indicating the influence of Al doping. Another interesting observation in the O 1s core spectrum was the presence of oxygen defect-related peaks. The peak at 530.23 eV suggests the presence of oxygen vacancies in the prepared CuO nanostructure,^34^ while the peak at 531.50 eV is attributed to the concentration of oxygen interstitials.^34^ These defect-related peaks were also observed in the PL spectra. The ratio of the areas of these defect peaks showed enrichment in O_i_ defects in the doped thin-film samples compared with the pristine samples. A similar trend was also observed in the PL spectra. Thus, both XPS and PL results suggest that Al doping results in the suppression of V_O_ defects.

The core spectrum in the binding energy region of 70–87 eV was obtained in order to study the Al 2p peaks. However, this binding energy region also includes the Cu 3p peaks, as observed from the corresponding spectrum shown in Fig. 10d. When the dopant concentration was very small, the signal corresponding to the Al 2p level was overpowered by that of the Cu 3p level. The Cu 3p doublets were observed at 75.71 and 78.07 eV along with their satellite peaks located at higher binding energy values of 84.37 and 85.54 eV, respectively. The observed peak positions match those observed in similar reports.^35^ The deconvolution of the spectra revealed the presence of Al 2p peaks at around 75.74 eV, confirming the integration of Al^3+^ at the Cu sites in CuO.^36^ The doublet of Al 2p could not be separated by deconvolution because the doublet peaks are only separated by 0.44 eV,^37^ which is lower than the resolution of the Al Kα X-ray source. The increased FWHM and the slight shift in the peak position of Al 2p are also due to the overlapping of the Al 2p_1/2_ and Al 2p_3/2_ peak signals.

The elemental composition of CAO 1 film was calculated from XPS analysis using the formula,^38^9
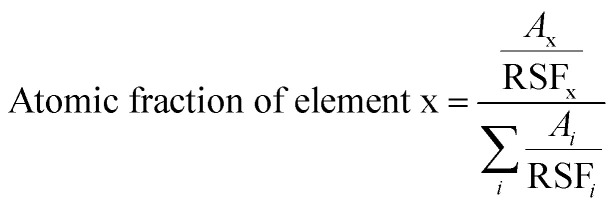
where RSF is the relative sensitive factor and *A* is the area of the corresponding peak. It was observed that the film was oxygen-rich in nature, with 57% oxygen, 42% Cu and 0.9% Al. These results are comparable with those obtained from EDS.

### 3.7 Electrical properties

**Fig. 11:**
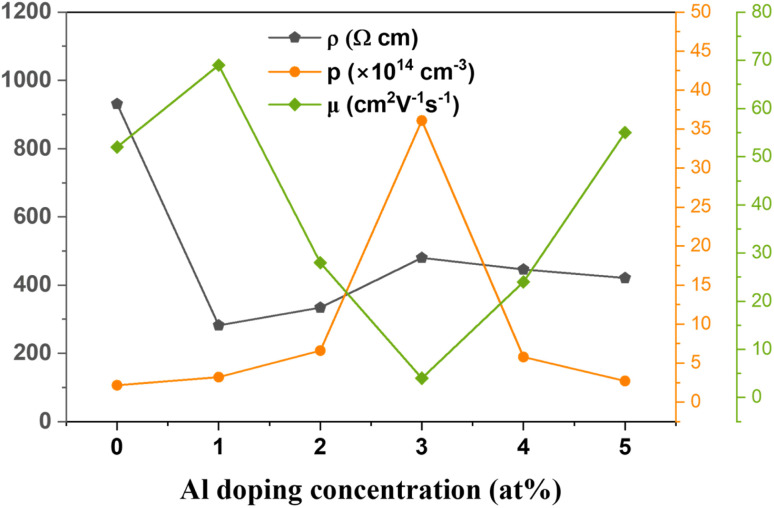
Variation in the electrical properties of the synthesized CAO films.

The electrical properties of the prepared CAO thin films were obtained from Hall measurements in the van der Pauw configuration. The variations in the electrical properties, including resistivity (*ρ*), mobility (*μ*) and carrier density (*p*), of the CuO thin films with Al doping are shown in Fig. 11. All the films were p-type in nature due to the presence of *V*_Cu_. Al-doped films showed a higher number of charge carriers than the undoped CuO films.

The carrier density increased from 2.17 × 10^14^ cm^−3^ in undoped films to 36.10 × 10^14^ cm^−3^ in 3 at% Al-doped CuO thin films. However, a further increase in doping resulted in a decrease in the number of charge carriers. The carrier mobility of the films showed an inverse relationship with the carrier density. The mobility of charge carriers was as high as 69 cm^2^ V^−1^ s^−1^ at low levels of doping (1 at%), and when the doping concentration was further increased, carrier mobility decreased. The enhanced mobility can be due to the increased crystallite size in the samples after doping. Though the 3 at% Al-doped sample showed a great increase in the number of charge carriers, due to the reduction in crystallite size, these additional charges would increase the scattering at the grain boundaries and also among themselves. Hence, the resistivity of this sample was the highest among the doped films. For the efficient performance of a solar cell, the absorber layer should have low resistivity and high mobility. Thus, Al doping has a positive impact on the electrical properties of CuO thin films by effectively improving their conductivity, thereby making them suitable for use in solar cells as absorber layers.

### 3.8 Device simulation

**Fig. 12:**
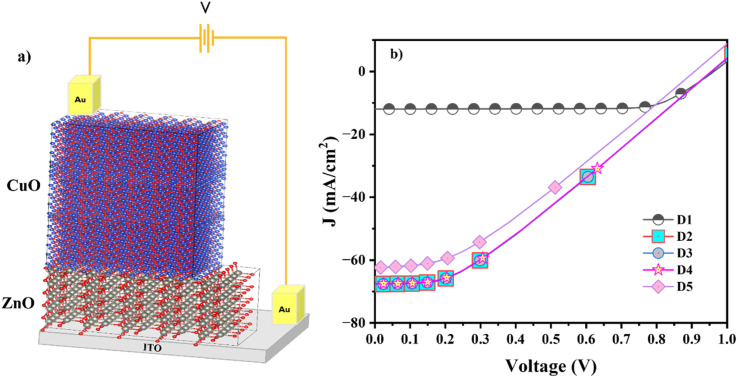
(a) Schematic of the device structure. (b) Simulated plots of the current density *vs.* voltage of devices under illumination.

To evaluate the potential of CuO and Al-doped CuO as absorber layers in solar cells, it is imperative to study the photovoltaic properties of heterostructures fabricated based on these films. Solar cells require a p–n junction heterostructure made of a window layer and an absorber layer. The suitable selection of substrate, window layer and contacts determines the efficiency of the heterostructure. The window layer is usually a large bandgap semiconductor with high transparency to allow the sunlight to reach the junction. Some commonly used window layers in solar cells are TiO_2_, ZnO, CdS, *etc.* Among these, oxide-based solar cells are preferred due to their ease of availability, non-toxicity, atmospheric stability and low production cost. Even though ZnO and TiO_2_ have ideal bandgaps for a window layer, for CuO with a monoclinic lattice (*a* = 4.5 Å, *b* = 3.3 Å and *c* = 5.1 Å), ZnO with a hexagonal lattice (*a* = 3.2 Å and *c* = 5.2 Å) has a greater lattice match. This reduces the probability of defect formation and thereby carrier recombination at the interface. In this study, we have used ITO as the substrate and ZnO as the n-type window layer with gold contacts. The schematic representation of the heterostructure is given in Fig. 12a.

Many software packages, such as wx-AMPS, AMPS 1D, and SCAPS-1D, are available for the numerical simulation of heterojunction solar cells. Among these, the most used and user-friendly is SCAPS-1D, which was designed by Marc Burgelman *et al.*^13^ for the real-time simulation of the electrical characteristics of heterojunction solar cells. This simulation tool helps us understand the basic principles of solar cells and the key factors affecting their performance. SCAPS 1D software solves one-dimensional differential equations that influence the conduction mechanism of charge carriers in a semiconductor in its stable state. In this work, the modelled ITO/ZnO/CuO solar cells were simulated using SCAPS-1D software in solar AM 1.5 illumination. Here, we studied the performance of CuO, 1 at% Al doped CuO solar cells and the influence of O_i_ defects concentration on the efficiency of 1 at% Al doped CuO solar cells. The basic input parameters of individual layers of each device used for simulation are listed in Table 4.

**Table 4:** Numerical parameters of the layers used in device simulation

Material properties	n-ITO	p-CuO	n-ZnO
Thickness (nm)	500	1000	100
Band gap (eV)	3.5	1.47	3.3
Electron affinity (eV)	4	4.18	4
Relative dielectric permittivity	9	15.6	9
CB effective density of states (cm^−3^)	2.2 × 10^18^	1.98 × 10^17^	3.7 × 10^18^
VB effective density of states (cm^−3^)	1.8 × 10^19^	6.5 × 10^20^	1.8 × 10^19^
Electron mobility (cm^2^ V^−1^ s^−1^)	20	220	100
Hole mobility (cm^2^ V^−1^ s^−1^)	10	12	25
Shallow uniform donor density *N*_D_ (cm^−3^)	1 × 10^21^	0	1 × 10^18^
Shallow uniform acceptor density *N*_A_ (cm^−3^)	0	10^16^	0

The simulated *J*–*V* characteristics of the devices are given in Fig. 12b, and the obtained cell parameters are tabulated in Table 5. All the devices showed very high efficiency, and the maximum efficiency of 20.97% was shown by the D4 sample. D1 with undoped CuO as the absorber layer showed an efficiency of 8.67%. When Al-doped CuO was used instead of CuO as the absorber layer, the cell efficiency improved to 20.85% mainly due to the improved *J*_sc_ value. This is in line with the experimental results, which revealed an increase in carrier density after doping. However, the FF of the cell decreased considerably. This indicates a significant rise in internal losses. Further, after doping, the shunt resistance decreased, indicating that the internal loss was due to low shunt resistance. As *V*_oc_ remained the same before and after doping, there was no significant change in the fundamental voltage generation capability of the cell. Hence, the key reason for improved efficiency is the substantial increase in *J*_sc_. This suggests a major improvement in the ability of the cell to generate current, likely outweighing the negative impact of the decreased fill factor on overall efficiency. In simpler terms, even with more internal losses due to lowered shunt resistance, the much greater number of carriers generated (reflected by *J*_sc_) leads to a net gain in efficiency.

**Table 5:** Device parameters obtained from SCAPS 1D simulations

Sample name	Device	O_i_ defect concentration (cm^−3^)	*V*_oc_ (V)	*J*_sc_ (mA cm^−2^)	FF (%)	*η* (%)
D1	ITO/ZnO/CuO	1 × 10^12^	0.96	12	75	8.67
D2	ITO/ZnO/CAO 1	—	0.96	68	32.15	20.85
D3	ITO/ZnO/CAO 1	1 × 10^12^	0.96	68	32.15	20.85
D4	ITO/ZnO/CAO 1	1 × 10^14^	0.96	68	32.43	20.97
D5	ITO/ZnO/CAO 1	1 × 10^16^	0.91	62	34.07	19.36

The influence of O_i_ defects on the performance of Al-doped CuO solar cells can be understood from the obtained results. The obtained values show that the concentration of O_i_ defects plays a pivotal role in improving the efficiency of Al-doped solar cells. The efficiency remained the same at low concentrations of defects, whereas the efficiency decreased at higher doping levels. At a defect concentration of 1 × 10^14^ cm^−3^, the efficiency improved slightly from 20.85 to 20.97% and then decreased with a further increase in concentration. The improved efficiency is associated with the improved *R*_s_ and *R*_sh_ resistance values, which improve the FF and therefore, overall efficiency. Hence, it is possible to tune the efficiency of a solar cell by controlling the O_i_ defects. Hence O_i_ defects, as well as Al doping, play a significant role in improving the performance of CuO solar cells.

## 4. Conclusions

Thin films of CuO and Al-doped CuO were synthesized on glass substrates *via* the spray pyrolysis method. The impact of Al doping on the inherent defects in CuO films was comprehensively investigated by analyzing the structural, optical, and electrical properties of the sprayed films. Al doping was found to increase the O_i_ defects in the CuO thin films, as confirmed by PL and XPS analyses. This increase in O_i_ defects resulted in higher charge carrier concentrations, consequently reducing the electrical resistivity of the films. The experimental findings are supported by DFT studies, which identified O_i_ as the most viable and stable defect compared with others. This discovery addresses current concerns in CuO thin-film solar cell research. Moreover, besides the enhancement in electrical properties, the improved structural and optical characteristics make these Al-doped thin films highly suitable for application as absorber layers in solar cells. SCAPS simulation studies further confirmed this potential, suggesting a potential increase in cell efficiency by up to approximately 20.97%.

## Data availability

The data that support the findings of this study are available from the corresponding author, mahesha.mg@manipal.edu, upon reasonable request.

## Author contributions

Adithya Prakash: conceptualization, investigation, formal analysis, methodology, visualization, writing – original draft. Vikash Mishra: methodology (DFT calculations). Mahesha M G: conceptualization, supervision, writing – review & editing.

## Conflicts of interest

Authors have no conflict of interest to declare

## Supplementary Material

RA-014-D4RA06413E-s001
