# Supplementary material for: Probing intrinsic defects of aluminium-doped CuO thin films for solar cell applications
Source: RSC Adv. 2024 Nov 5;14(47):35184–97. doi: 10.1039/d4ra06413e (PMC11536046; doi:10.1039/d4ra06413e)
Supplement: RA-014-D4RA06413E-s001 [file RA-014-D4RA06413E-s001.pdf]

Supplementary Information (SI) for RSC Advances.
This journal is © The Royal Society of Chemistry 2024

Probing Intrinsic Defects Developed in Aluminium Doped CuO Thin Films for Solar Cell Applications

Adithya Prakash, Vikash Mishra and Mahesha M G *

Department of Physics, Manipal Institute of Technology, Manipal Academy of Higher Education 576104, India

*Corresponding Author.

Email: mahesha.mg@manipal.edu

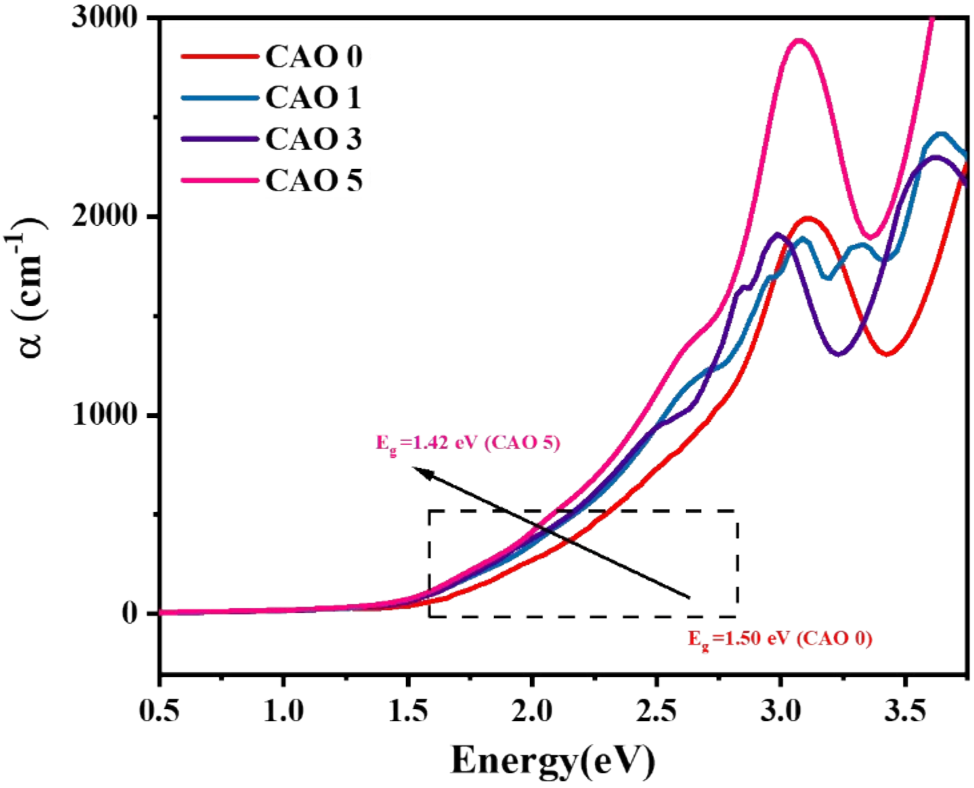

Fig S1: Simulated optical absorption spectra of Al doped CuO.

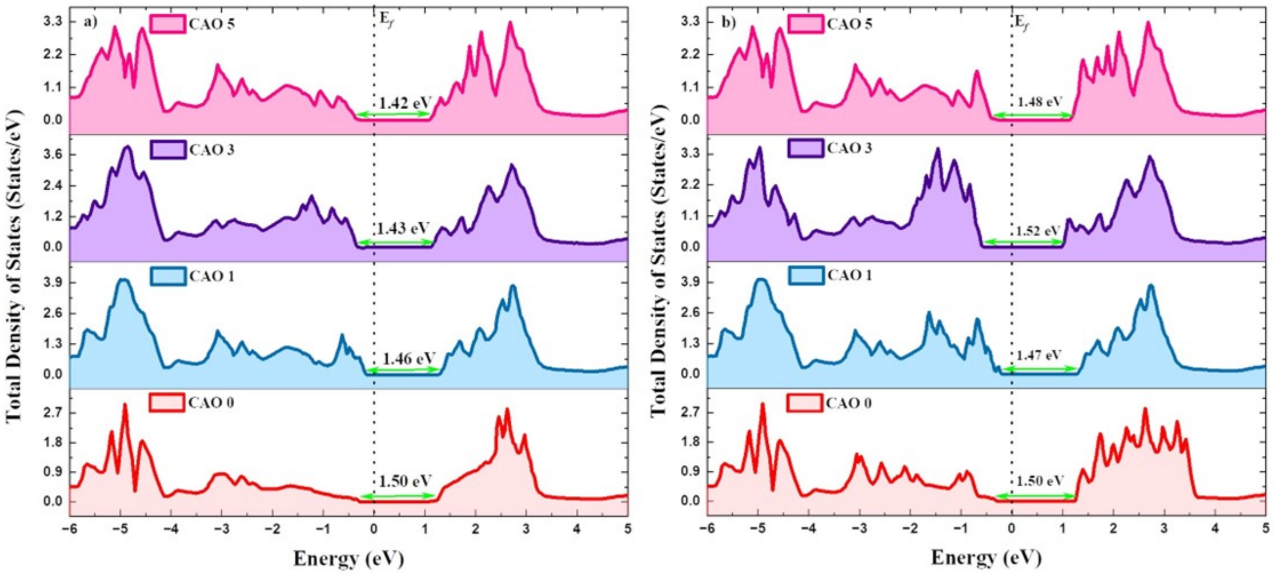

Fig S2: Total density of states of CAO 0, CAO 1, CAO 3 and CAO 5 a) without defects and b) with O_i defects.
